# Philadelphia Obstetrical Society

**Published:** 1890-01

**Authors:** J. M. Baldy

**Affiliations:** Secretary


### PHILADELPHIA OBSTETRICAL SOCIETY.

---

NOVEMBER 7, 1889.

THEOPHILUS PARVIN, M. D., in the chair.

DR. J. M. BALDY reported a case of vaginal cyst which had occupied the anterior portion of the anterior vaginal wall. It had prolapsed like a cystocele for years, and had been taken and treated for such. He had enucleated the cyst without much trouble. He called attention to the various theories as to the origin of these cysts, and came to the final conclusion that they arose from the vaginal glands, as retention cysts. Although it was claimed by some that vaginal glands did not exist, yet he thought that the positive evidence in favor of their existence, produced by such men as Dubois, Klek, Veit, Von Preuschen and others, settled the question in the affirmative. He thought the position of these cysts at times proved conclusively that those individual tumors could not have arisen from the ducts of Gärtner. Where the cysts were small or of moderate size, he preferred to enucleate them, even though this measure had been pronounced difficult and dangerous. He had not found it either one or the other. Accidents might happen in the hands of the clumsy, but not in the hands of a surgeon.

DR. DACOSTA thought that the method of enucleation the typical one, but that there were cases in which this operation was not feasible. He quoted such a case. Sometimes simple incision was sufficient. In others he had cut the top away in an oval shaped piece, leaving the bottom of the cyst against the anterior vaginal wall. This operation had served him well.

DR. E. E. MONTGOMERY exhibited the specimen of a supra-vaginal hysterectomy. The tumor had grown for eight years. The patient had never been pregnant. There was no great amount of hemorrhage. The suffering was from the pressure. Incision was five inches. The broad ligaments were grasped in forceps, and cut so as to allow them to retract from the tumor, thus making a good pedicle. Transfixion pins were passed through the stump, and it was secured by a serre noeud and fastened in the lower angle of the wounds. Irrigation of the abdomen was made, and a drainage-tube was introduced. The stump was dusted with iodoform, and covered with gauze. The stump came away in a week, and the patient made a good recovery.

DR. JOSEPH PRICE exhibited a large pus-tube and ovarian abscess. The husband had had gonorrhœa. The specimen demonstrated multiple abscess in the pelvis, and the folly of treatment by vaginal puncture. He had seen two other cases of gonorrhœal salpingitis in the past seven days. Dr. Price also exhibited a large fibroid tumor of the uterus removed successfully by supra-vaginal hysterectomy. The tumor had been treated three times a week for eleven weeks, by a pupil of Apostoli, with electricity. There was less hemorrhage but a great increase in watery discharge, together with rapid enlargement of the tumor, increase of pressure symptoms, and rapid decline in general health of the patient. The case was complicated by a small ovarian cyst on the left side.

DR. G. BETTON MASSEY had always seen fibroid tumors, unless cystic, get harder and smaller under the use of electricity. He had always seen lessening of hemorrhage and coincident improvement in health. He was not in favor of puncture, although he had seen some good result from it.

DR. JOSEPH HOFFMAN said that this differential diagnosis of the complications of fibroid tumors (cystic from true fibroids, etc.) was just the trouble. Keith, in his long, successful series, was in the great majority of them uncertain of his diagnosis, and yet the electricians could always make this diagnosis.

DR. J. PRICE said that the appendages should be removed while the tumor was small. The results of this operation were wonderfully good. The deaths following the electrical treatment are as many as those following hysterectomy, and the results are not to be compared, In the one all the symptoms come back; in the other the disease is completely removed.

DR. WM. GOODELL read the history of a case of Burst Purulent Appendages. Before Dr. Goodell had seen patient she had had an attack of general peritonitis, supposed by her physician to be due to a burst cyst. She was six weeks in bed. On October 9th, Dr. Goodell found the womb fixed and a tumor on the side, which suddenly collapsed while he was examining it. Two days later he operated on her, and removed from the peritoneum a large amount of grumous and dirty fluid. It had come from a collapsed right ovarian cyst. Both tubes and the other ovary contained the same fluid. Recovery was prompt.

DR. M. PRICE had elsewhere called attention to the danger of rupturing cysts by rough handling, especially extra-uterine gestation sacs. He thought that Dr. Goodell was very lucky in saving his patient. Where such an accident has happened the operation should be done at once, and a delay of two days was not proper.

DR. B. F. BAER had met with several cases of cysts which were ruptured. In one case he had found a cyst and advised its removal; subsequently another physician said there was no cyst there. She then returned to Dr. Baer and he could not find the tumor even under ether. A few months later the tumor had reappeared and a successful operation removed it.

DR. WM. GOODELL could not wholly accept the remarks of Dr. Price. He had some years ago accidentally ruptured a small cyst while examining patient on his office table. It returned, and twice subsequently he ruptured it. After the third time it did not return. He afterwards did the same thing in another case. At the present day he would not do so; but these facts showed the tolerance of the peritoneum to such accidents.

J. M. BALDY, *Secretary.*

